# Copy number increases of transposable elements and protein‐coding genes in an invasive fish of hybrid origin

**DOI:** 10.1111/mec.14134

**Published:** 2017-04-28

**Authors:** Stefan Dennenmoser, Fritz J. Sedlazeck, Elzbieta Iwaszkiewicz, Xiang‐Yi Li, Janine Altmüller, Arne W. Nolte

**Affiliations:** ^1^ Department for Evolutionary Genetics Max‐Planck Institute for Evolutionary Biology Plön Germany; ^2^ Institute for Biology Carl von Ossietzky University Oldenburg Oldenburg Germany; ^3^ Department of Computer Science Johns Hopkins University Baltimore MD USA; ^4^ Department of Evolutionary Biology and Environmental Studies University of Zurich Zurich Switzerland; ^5^ Cologne Center for Genomics, and Institute of Human Genetics University of Cologne Cologne Germany

**Keywords:** array‐comparative genomic hybridization, Cottidae, digital droplet PCR, hybrid speciation, invasion genetics, structural mutations

## Abstract

Evolutionary dynamics of structural genetic variation in lineages of hybrid origin is not well explored, although structural mutations may increase in controlled hybrid crosses. We therefore tested whether structural variants accumulate in a fish of recent hybrid origin, invasive *Cottus*, relative to both parental species *Cottus rhenanus* and *Cottus perifretum*. Copy‐number variation in exons of 10,979 genes was assessed using comparative genome hybridization arrays. Twelve genes showed significantly higher copy numbers in invasive *Cottus* compared to both parents. This coincided with increased expression for three genes related to vision, detoxification and muscle development, suggesting possible gene dosage effects. Copy number increases of putative transposons were assessed by comparative mapping of genomic DNA reads against a de novo assembly of 1,005 repetitive elements. In contrast to exons, copy number increases of repetitive elements were common (20.7%) in invasive *Cottus*, whereas decrease was very rare (0.01%). Among the increased repetitive elements, 53.8% occurred at higher numbers in *C. perifretum* compared to *C. rhenanus*, while only 1.4% were more abundant in *C. rhenanus*. This implies a biased mutational process that amplifies genetic material from one ancestor. To assess the frequency of de novo mutations through hybridization, we screened 64 laboratory‐bred F_2_ offspring between the parental species for copy‐number changes at five candidate loci. We found no evidence for new structural variants, indicating that they are too rare to be detected given our sampling scheme. Instead, they must have accumulated over more generations than we observed in a controlled cross.

## Abstract 2

see also the Perspective by Choudhury and Parisod

## 1 Introduction

Hybridization can be an important driver of evolutionary change and may lead to homoploid hybrid speciation of animals and plants (Abbott et al., 2013; Mallet, 2007; Nolte & Tautz, 2010; Schumer, Rosenthal, & Andolfatto, 2014). It remains challenging to identify genetic factors that determine hybrid fitness, partly because of their complex genomic basis (Landry, Hartl, & Ranz, 2007). Reduced hybrid fitness can be explained through Bateson–Dobzhansky–Müller incompatibilities of alleles (Bateson, 1909; Dobzhansky, 1937; Müller, 1942) that can increase reproductive isolation and hamper the evolution of admixed lineages (Maheshwari & Barbash, 2011). On the other hand, hybridization can lead to new combinations of allelic variants and therefore be a source of evolutionary novelty and ecological niche divergence (Lexer, Lai, & Rieseberg, 2003). A common explanation is given by the process of transgressive segregation, which is caused by novel interactions of alleles that evolved in isolation and subsequently segregate in the hybrid offspring (Rieseberg, 1997; Rieseberg, Archer, & Wayne, 1999). This is plausible because hybridization introduces a wealth of genetic diversity that can affect evolutionary processes during the initial and highly dynamic phase of hybrid speciation.

In addition to effects caused by parental alleles, an increased rate of de novo structural mutations in hybrids appears possible, similar to the finding of increased point mutations in heterozygotes (“heterozygosity instability”; Amos, 2010; Xie et al., 2016). Whether this actually occurs and leaves detectable genetic signatures has received comparatively little attention in studies on hybrid speciation. However, it has already been found that hybrid crosses may exhibit de novo copy‐number variation that is frequent enough to be observed among siblings of the same litter of mice (Scavetta & Tautz, 2010). Although copy‐number variations (CNVs) may infer fitness costs associated with gene dosage alteration (Orozco et al., 2009; Papp, Pal, & Hurst, 2003), they may also contribute to the acquisition of new adaptive functions and promote species diversification (Kondrashov, 2012; Machado et al., 2014). Hybridization can also increase mutation rates through the activation of transposable elements (TEs), which have been reported to undergo “bursts of transposition” in hybrids of plants (McClintock, 1984; Renaut, Rowe, Ungerer, & Rieseberg, 2014; Ungerer, Strakosh, & Stimpson, 2009) and animals (García Guerreiro, 2015; Labrador & Fontdevila, 1994; O'Neill, O'Neill, & Graves, 1998; Renaut, Nolte, & Bernatchez, 2010). Transposition events can have numerous genomic consequences, including copy‐number changes of protein‐coding genes (Vogt et al., 2014; Xing et al., 2006), disruption of genes or regulatory motifs (Bonchev & Parisod, 2013) or the introduction of inversion break points that may negatively affect genomic stability (Casals, Cáceres, & Ruiz, 2003; Symer et al., 2002). Accordingly, increased transposition is often detrimental and therefore quickly silenced by the host genome (Castel & Martienssen, 2013). Conversely, transposons can also create adaptive genetic variation and be subject to positive natural selection (Casacuberta & González, 2013; Chuong, Elde, & Feschotte, 2016). Similar to copy‐number variation of genes, this has been repeatedly suggested to promote speciation and adaptive radiations (Belyayev, 2014; Fontdevila, 2005).

The expected number of gene and transposon copies in a lineage of hybrid origin should correspond with the average of the copy number in the parental species (e.g., Kawakami, Dhakal, Katterhenry, Heatherington, & Ungerer, 2011; Scavetta & Tautz, 2010). Deviations from this average would be conceivable if the genetic contributions from both parent species to the hybrid gene pool are not even. Copy numbers may, however, not exceed observed parental averages due to admixture alone. Instead, copy‐numbers outside the parental values could indicate the evolution of new copies and be a distinguishing feature of hybrid lineages. If rapidly evolving structural variants were also subject to positive selection, they could be among the first to contribute to adaptive evolution of hybrid lineages. Although the underlying mutational mechanisms are known from controlled hybrid crosses (e.g., García Guerreiro, 2014; McClintock, 1984; Scavetta & Tautz, 2010), few empirical studies have documented the evolutionary dynamics of copy‐number variation and transposition in natural admixed lineages. For example, studies on homoploid sunflower hybrid species revealed a massive accumulation of Ty3/gypsy‐like TEs during the past 0.5–1 million years (Renaut et al., 2014; Ungerer et al., 2009). On the other hand, transposition bursts have not been observed in F_1_ hybrid sunflowers or contemporary admixture zones between their parental species (Kawakami et al., 2011; Renaut et al., 2014). Consequently, the relative importance of short‐term compared to long‐term evolutionary processes that drive the proliferation of TEs remains unclear when hybrid lineages are ancient.

Here, we analysed whether CNVs have increased in a small freshwater fish of hybrid origin that has emerged postglacially, possibly within the last 200 years (Nolte, Freyhof, Stemshorn, & Tautz, 2005). The so‐called invasive *Cottus* provides a unique natural setting to explore evolutionary mechanisms that may initiate the evolution of a homoploid hybrid species (Nolte & Tautz, 2010). Invasive *Cottus* is a lineage of hybrid origin that has colonized lower reaches of rivers and streams of intermediate size, whereas the parental species *Cottus perifretum* and *Cottus rhenanus* live in parapatry, apparently confined to the headwaters of smaller tributaries (Nolte et al., 2005; Stemshorn, Reed, Nolte, & Tautz, 2011). A likely explanation for the success in lower reaches of rivers is that invasive *Cottus* has evolved new beneficial traits through hybridization that are driving divergence between invasive *Cottus* and its parental species. Besides barriers to gene flow in hybrid zones (Nolte, Freyhof, & Tautz, 2006; Nolte, Gompert, & Buerkle, 2009), this manifests in patterns of gene expression that are unique to invasive *Cottus* (Czypionka, Cheng, Pozhitkov, & Nolte, 2012). In this study, we tested whether rapid copy‐number evolution of protein‐coding genes and TEs occurs in natural populations of invasive *Cottus*. We expected to find overall more gene duplications than deletions (e.g., Katju & Bergthorsson, 2013), as well as an increased variance of copy numbers compared to the parental species (Scavetta & Tautz, 2010). To further explore the possibility that hybridization causes rapid evolution of de novo structural variants, we tested laboratory‐raised F_2_ (selfed F_1_) *C. rhenanus* × *C. perifretum* crosses for copy‐number changes of candidate genes and transposons that have increased in copy number in natural populations of invasive *Cottus*. Finally, we searched for associations between changes in gene copy number and gene expression in invasive *Cottus* to explore possible contributions of CNVs to phenotypic change.

## 2 Materials and Methods

### 2.1 Sample collection and DNA extraction

Samples were collected in Northern Germany (watersheds of rivers Sieg, Wied, Emscher, Mosel, Lippe, Ruhr, Wupper, Wisper), Belgium (River Scheldt watershed) and Great Britain (watersheds of rivers Wensum, Great Ouse) between 2001 and 2015 (Table 1). Gender was determined by carefully inspecting gonad development. Genomic DNA was extracted from blood samples, using the 5 prime ArchivePure DNA Tissue kit (5 PRIME, Hamburg, Germany) or the Qiagen Blood and Tissue kit. Extracted DNA was quality checked for the presence of high molecular weight genomic DNA by agarose gel electrophoresis.

**Table 1:** Individual samples used for comparative genomic hybridization array (aCGH) microarrays and genome mapping. Indicated are number, gender and geographic coordinates for each population

Population	aCGH	Genome mapping	Lat./Long.
*Cottus rhenanus*			
Bröl (Sieg)	1f^a^	1	50°47′43″N 7°20′22″E
Mengbach (Sieg)	2f	1f	50°46′57″N 7°25′13″E
Naafbach (Sieg)	1m^a^	1	50°52′00″N 7°16′00″E
Krabach (Sieg)	1m, 1f	1m	50°45′45″N 7°24′47″E
Wahnbach (Sieg)	1m	1m	50°50′50″N 7°19′15″E
Ottersbach (Sieg)		1m	50°46′32″N 7°28′56″E
Fockenbach (Wied)	1m	1	50°32′14″N 7°25′46″E
Boye (Emscher)		1m	51°33′33″N 6°55′42″E
Kyll (Mosel)		1	50°14′00″N 6°39′00″E
Soestbach (Lippe)		1m	51°34′45″N 8°05′17″E
Wisper (Rhein)		1	50°20′58″N 7°50′60″E
Wupper (Rhein)		1	51°15′22″N 7°16′19″E
Wanne (Ruhr)		1m	51°25′29″N 8°03′05″E
*Cottus perifretum*			
Larse Beek (Schelde)	2f^a^	1	51°17′10″N 4°30′11″E
Witte Nete (Schelde)	3m^a^, 1f^a^	1	51°14′22″N 5°4′11″E
Zwanebeek (Schelde)	2	1	51°14′44″N 4°34′58″E
Trouille (Schelde)		1	50°23′55″N 4°0′52″E
Molenbeek (Schelde)		1	50°48′14″N 3°40′42″E
Twin (Great Ouse)		1	51°58′10″N 0°57′59″E
Wensum (Yare)		1	52°42′59″N 1°1′59″E
*Cottus “invasive”*			
Sieg (Rhein)	4m, 4f	5m, 2f, 3	50°47′55″N 7°10′45″E

Stream name with higher order river given in parentheses.

m = male, f = female.

a Laboratory‐bred.

### 2.2 Array‐CGH design and calibration

We developed a custom 60‐mer oligonucleotide comparative genomic hybridization array (aCGH) to screen for copy number differences across 10,979 genes that could be annotated to the threespine stickleback genome (*Gasterosteus aculeatus;* BROADS1.56). Briefly, an aCGH experiment compares genomic DNA of test and reference samples that are digested (optional), colour‐labelled (Cy3; Cy5) and hybridized together on an array that is scanned for fluorescence intensity values. The presence of gene duplications or deletions is inferred from log2‐intensity ratios between test and reference signals.

Probe design for the microarrays was largely based on *Cottus* expressed sequences (ESTs) assembled with the trinity software (Grabherr et al., 2011). For the transcriptome assembly, total RNA was extracted from different tissues (gills/heart, liver, fins/skin, digestive tract, head without gills, white muscle) of a *C. rhenanus* male from Fockenbach using a TRIzol protocol (Invitrogen, Carlsbad, CA, USA). Libraries were prepared with the Illumina Truseq Kit and sequenced from two sides (100‐bp read length) on an Illumina GAIIx sequencer. We only targeted genes that could be annotated to the threespine stickleback genome, aiming for a coverage of one probe per exon (“exon‐array CGH”; Dhami et al., 2005). Additionally, we included probes for genes that were identified to be either upregulated or downregulated in invasive *Cottus* in a previous gene expression microarray study (Czypionka et al., 2012). We first conducted a blat search (blat version 35x1; Kent, 2002; *e*‐value < 10^−3^) of *Cottus* ESTs against the stickleback genome and subsequently used best matching sequences larger than 60 bp for a second blat search against stickleback coding sequences (retrieved from the biomart database version 0.7; www.ensembl.org/biomart). The sense direction of all probe sequences was inferred from the directionality given in the blat results. Sequences identified as negative strands were reverse‐transcribed, and sequences with contrasting directionality information between the ensemble database and the blat results were removed.

To optimize probe selection and to validate the performance of probes, we initially performed a microarray calibration experiment (Czypionka et al., 2012; Pozhitkov, Noble, Bryk, & Tautz, 2014). We designed three probes per exon, which were selected according to an optimal melting temperature of 80°C. Probe sequences were submitted to SureDesign (www.agilent.com/genomics/suredesign) for the design of a SurePrint G3 custom CGH microarray (8x60k) with two replicates per probe and the addition of a 15‐bp linker recommended by Agilent. A dilution series of eight starting DNA concentrations ranging between 0.0625× and 4× of a recommended starting DNA amount of 400 ng (corresponding to 25, 50, 100, 200, 400, 800, 1,200, and 1,600 ng) was prepared for a single *C. rhenanus* and a single *C. perifretum* individual. Both dilution series (*C. rhenanus*, Cy3 labelled; *C. perifretum*, Cy5 labelled) were cohybridized on the calibration array. Linear regressions on average (of two probe replicates) signal intensities across the dilution gradient were fitted for red (Cy5) and green (Cy3) channels separately. Next, we excluded probes with nonlinear response curves (*R* ^2^ < .95), a signal‐to‐error ratio below 10, as well as unresponsive probes. From the remaining probe set, we retained one probe per gene by filtering for a signal intensity increase (regression slope) being closest to one. The final probe set contained 12,105 probes covering 10,979 stickleback genes, as well as 446 candidate probes from Czypionka et al. (2012) (see Table S3). The final probe set was sent to SureDesign for the construction of an experimental 8x60k array with five replicates per probe, and a 15‐bp linker added to each probe as recommended by Agilent (SureDesign‐ID: 070467).

The experimental array was used for a common reference aCGH experiment. Three array slides were used to cohybridize 24 test samples (eight individuals from each of *C. rhenanus, C. perifretum,* and invasive *Cottus*; all Cy5 labelled) with a common reference sample (a female individual of *C. perifretum* from Larse Beek; Cy3 labelled) (Table 1). Samples were randomly assigned to the 24 arrays, and for each sample, genomic DNA was quantified using a fluorescence NanoDrop (dsDNA BR Assay Kit, Thermo Fisher Scientific, Waltham, MA, USA) to achieve equal amounts (400 ng) of starting DNA. For all aCGH experiments, genomic DNA was labelled following the “direct method” as outlined in the Agilent protocol “Agilent Oligonucleotide Array‐based CGH for genomic DNA analysis” (version 7.3, March 2014; Agilent Technologies). The same protocol was used for subsequent hybridization and washing of the arrays. Microarrays were covered with Agilent ozone‐barrier slide covers and scanned using an Agilent Microarray Scanner (type C). Signal intensity values were extracted using the Agilent feature extraction software version 10.7.3.1.

### 2.3 Quality filtering, normalization

Quality control metrics provided in the feature extraction output files were carefully inspected for the spatial distribution of outliers on the arrays. A pipetting error before hybridization created a noticeable bubble in the centre of nine experimental arrays, resulting in decreased signal intensity values and clustering of outliers in these regions. We therefore decided to remove all data affected by such bubble artefacts (~12% of 1,452,600 probe replicates present on 24 arrays). For this purpose, we first retrieved coordinates for individual probe replicates (“features”) from the feature extraction output files and plotted the spatial distribution of log‐signal intensities for each array (using the “levelplot” function in the r package latticeextra). We used the r package “splancs” (version 2.01‐36; Bivand & Gebhardt, 2000) to extract the coordinates of features located within bubble artefacts by drawing polygons around the bubbles, which were clearly visible through reduced log‐signal intensities. Coordinates of all features located within bubble areas were then used to remove affected data from the complete data set. Next, we excluded outliers among probe replicates as determined by signal intensities exceeding the 1.5× interquartile range and took the average of the remaining probe replicates for further downstream analyses. The final data set covered 12,102 probes represented on average on 22 out of 24 arrays.

To account for probe‐specific binding coefficients in the observed signal intensities, we applied a calibration on average signal intensities as recommended by Pozhitkov et al. (2014). For this purpose, we used the probe‐specific linear regression parameters (slope, intercept) from the calibration array, to estimate a calibrated starting DNA value (*DNAcal*) for each channel (Cy3, Cy5), calculated as *DNAcal = (observed signal *−* intercept)/slope*. Although this procedure reduces artefacts introduced through probe‐specific differences in binding behaviour, it does not remove intensity effects, which are visible in a “MA”‐plot as increasing log2‐(Cy5/Cy3)‐ratios (“M”) with increasing average (log‐) signal intensities (“A”). To remove such intensity effects and to normalize log2‐ratios to an average of zero, we performed a LOESS normalization (span = 0.3, iterations = 4) without background correction (Zahurak et al., 2007), as implemented in the r package limma (version 3.22.4, Ritchie et al., 2015). This method fits a smoothing curve based on locally weighted least‐squares regression and uses the residuals of the fitted curve as normalized log2‐ratios.

### 2.4 Array‐CGH data analyses and comparison to gene expression

Genes showing increased or decreased copy‐numbers in invasive *Cottus* compared to the (combined) parental species were identified using two‐sample Wilcoxon rank‐sum tests on normalized signal intensity values in r (FDR corrected for multiple testing; Benjamini & Hochberg, 1995). To test whether invasive *Cottus* show a higher tendency towards increased compared to decreased copy numbers, we tested for skewness of average log2‐ratios of invasive *Cottus* relative to parental *Cottus* log2‐ratios using a two‐sided D'Agostini test of skewness implemented in the r package “moments.” To test whether invasive *Cottus* showed an overall increase in variability of copy numbers, we conducted *t* tests (FDR corrected for multiple testing) to assess how many probes showed significantly higher variance of log2‐ratios in invasive compared to parental *Cottus*, and how often each of the parental species showed significantly higher variance compared to the other species.

Possible gene dosage effects of exon copy‐number changes in invasive *Cottus* were evaluated by comparing CNV candidates with lists of gene expression candidates showing significant higher expression in invasive *Cottus* compared to the parental species. These gene expression candidates were characterized in a previous microarray study using whole‐body tissues of juvenile *Cottus* individuals (Czypionka et al., 2012).

### 2.5 Genome sequencing, Repetitive element assembly and CNV analyses

We investigated the possibility of transposon accumulation in invasive *Cottus* by comparing the relative number of genomic reads that could be mapped to a de novo assembly of repetitive elements between invasive and parental *Cottus* species. For this purpose, we used whole‐genome Illumina paired‐end sequence reads of 13 *C. rhenanus*, seven *C. perifretum* and 10 invasive *Cottus* genomes (Table 1). Genomic DNA was fragmented using sonication (Covaris, Woburn, MA, USA), and fragments were end‐repaired and adapter ligated. The libraries were sequenced using an Illumina GAIIx sequencer with 2× 150‐bp paired‐end reads. Raw paired‐end Illumina GAII reads were quality trimmed using the software popoolation (Kofler et al., 2011). To facilitate the assembly process, sequence reads were first error‐corrected using the software lighter, with a k‐mer length of 31, a genome length of 1 Gbp, and a subsampling fraction of 0.1 (Song, Florea, & Langmead, 2014). For each individual, we created assemblies of putative TEs using a de Bruijn graph‐based approach implemented in the software tedna v. 1.2.2 (Zytnicki, Akhunov, & Quesneville, 2014). As parameter settings, we used a maximum graph size of 1,000, kmer 30, minimum size of 500 bp, and an insert size of 300 bp. To create an improved assembly with longer contigs, we assembled the resulting tedna output sequences in the software seqman pro version 2.0.0 (DNASTAR, Madison, WI, USA) using default settings. The consensus sequences were exported as fasta files and retained as a *Cottus* reference library of repetitive elements. To classify putative transposons, we used the software pasteclassifier (v. 1.0; Hoede et al., 2014). pasteclassifier searches for characteristic structural and functional features of TEs, including long terminal repeats (LTR), terminal inverted repeats (TIR), reverse transcriptases (RT), transposases, polyA tails and open‐reading frames (ORFs), and tests for similarities to known TEs provided by the repbase database (v. 20.04; Jurka et al., 2005), as well as to hidden Markov model‐based profiles of complete protein domains (pfam26.0_gypsydb; Finn et al., 2016). By combining all this information, pasteclassifier then classifies putative transposons to class and order level according to the Wicker hierarchical TE classification system (Wicker et al., 2007). Remaining unclassified sequences were further analysed using blastx searches in blast2go (Conesa et al., 2005).

To compare CNV of putative TEs among *Cottus* individuals, we mapped whole‐genome (Illumina) sequence reads (trimmed to 90‐bp length to ensure comparability among genomes) against the *Cottus* reference library of repetitive elements using the software nextgenmap with a minimum identity constraint of 95% (ngm version 0.4.12, Sedlazeck, Rescheneder, & von Haeseler, 2013). For each *Cottus* individual, we counted the number of mapped forward reads, divided it by the total number of reads that were used for mapping and multiplied it by one million. One‐sample Wilcoxon rank‐sum tests (FDR corrected for multiple testing) were then used to compare counts per million mapped reads between invasive and parental *Cottus* species.

### 2.6 Validation of candidate CNVs and screen for de novo CNVs in F_2_ crosses

We used digital droplet PCR (ddPCR; upgraded QX100 Droplet Digital PCR System; Bio‐Rad Laboratories, CA, USA) with EvaGreen dye as fluorescent marker to (1) validate a copy‐number increase of three genes and three repetitive elements for the same 24 individuals used for array‐CGH experiments and to (2) screen laboratory F_***2***_ crosses for de novo structural variants of ddPCR‐validated candidate CNVs. For the de novo structural variant screen in F_***2***_ crosses, we used 32 individuals from each of two *C. rhenanus* × *C. perifretum* families that were obtained by selfing F_1_ hybrids between individuals representing the ancestral species (Populations crossed: Bröl × Witte Nete and Naaf × Larse Beek, respectively).

To estimate copy numbers, we used multiplex reactions of the target sequence together with a single‐copy reference gene (McDermott et al., 2013; Miotke, Lau, Rumma, & Ji, 2014). As a reference single‐copy gene, we chose *rpl13a*, which has been used as a single‐copy housekeeping gene in threespine stickleback (Hibbeler, Scharsack, & Becker, 2008), and showed no signs of CNV in our own CGH‐array experiments. All primers were designed using primer3 (http://primer3.ut.ee) and together with the targeted regions visualized in igv Viewer (version 2.3.32, Thorvaldsdóttir, Robinson, & Mesirov, 2013) to ensure coverage by all individuals and to avoid common SNPs. Initial gradient PCRs for annealing temperature and different DNA concentrations were performed to optimize PCRs. For all PCRs, 20 μl of the mastermix was mixed with 70 μl Droplet Generation Oil (Bio‐Rad Laboratories) and partitioned into droplets using a QX100 Droplet Generator (Bio‐Rad Laboratories). For primer sequences as well as ddPCR cycling conditions, see Table S1. For all PCR products, fluorescence of individual droplets was measured using an upgraded QX100 Droplet Digital reader (Bio‐Rad Laboratories) and analysed with the quantasoft droplet reader software (Bio‐Rad Laboratories). To test for significant differences of copy numbers among species, we applied one‐way ANOVAs followed by Tukey HSD post hoc tests.

## 3 Results

### 3.1 Exon‐Array CGH copy‐number variation

**Figure 1:**
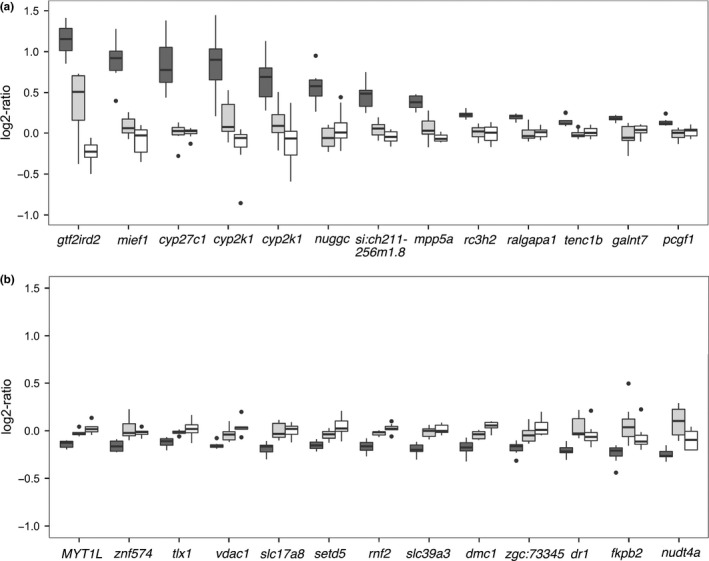
Candidate copy‐number variations genes from aCGH analyses with significantly increased (a) and decreased (b) log2‐ratios in invasive *Cottus* (dark grey = invasive *Cottus*, light grey = *C. perifretum*, white = *C. rhenanus*)

We compared CNV in eight invasive *Cottus* individuals with eight individuals of each of the parental species, *C. rhenanus* and *C. perifretum* to detect copy‐number changes of genes in invasive *Cottus*. Among 10,979 genes (exons) and additional 446 probes derived from a previous gene expression study (Czypionka et al., 2012), we identified 12 genes with increased as well as 13 genes with decreased copy numbers in invasive *Cottus* compared to both parental species (two‐tailed Wilcoxon rank‐sum test; FDR corrected; *p* < .05) (Figure 1; Table S2). Between the parental species *C. perifretum* and *C. rhenanus*, we found 1,920 genes (~15.9%) to differ in copy number (two‐tailed Wilcoxon test; FDR corrected; *p *< .05). Copy numbers did not differ significantly between males and females (results not shown).

In accordance with the general expectation to find more gene duplications than deletions, copy‐number variable genes in invasive *Cottus* showed a stronger signal of copy number increase than decrease (D'Agostini test: skew = 0.8918; *z* = 34.6861; *p* < .001). This trend of invasive *Cottus* to show more increases of copy numbers was also visible using arbitrary log2‐thresholds: Increased copy numbers were inferred for 409 genes that exceeded a log2‐ratio threshold of 0.5 (putative duplications), whereas decreased copy numbers were inferred for 314 genes falling below a log2‐ratio threshold of −0.5 (putative deletions) in at least one individual. Among those genes, 125 included individuals with signs of both deletions and duplications, leaving a total of 598 copy‐number variable genes (4.9%). When applying a less stringent log2‐ratio threshold of (±) 0.4, a total of 1346 (11.1%) of all genes were estimated to be copy number variable in at least one individual, which included 916 putative duplications and 733 putative deletions.

In contrast to our expectation, we found no increased variance of copy numbers in invasive *Cottus* compared to the parental species. Compared to *C. rhenanus*, the number of genes showing an increased variance was 825 in invasive *Cottus* and 947 in *C. perifretum*. *Cottus rhenanus* showed similar numbers of genes with increased variance compared to both other species: 769 genes were more variable than in *C. perifretum* and 685 genes more variable than in invasive *Cottus*. The variances between invasive *Cottus* and *C. perifretum* differed conspicuously from the above numbers in that differences between genes were comparably rare: invasive *Cottus* showed a higher variance than *C. perifretum* in 33 genes, whereas *C. perifretum* showed a higher variance than invasive *Cottus* in 36 genes.

### 3.2 Comparison of CNV candidates to gene expression

To explore possible gene dosage effects of increased exon copy numbers, we compared CNV candidate genes to lists of genes that were overexpressed in invasive *Cottus*. Possible gene dosage candidates included four probes (*GTF2IRD2, CYP27C1* and two probes for *CYP2K1*) that were previously found to be overexpressed in whole‐body tissues of juvenile individuals of invasive *Cottus* (Czypionka et al., 2012).

### 3.3 De novo assembly and copy‐number variation of repetitive elements

To assess a possible accumulation of TEs in invasive *Cottus*, we used a genome mapping approach to compare the relative number of reads mapped to a de novo assembly of repetitive elements. The de novo assemblies in tedna resulted in a total of 7,548 sequences, which were assembled in seqman pro into 1,005 contigs (hereafter referred to as “repetitive elements”; size range: 500‐50k bp; n50: 2,978 bp; Table S4). Using pasteclassifier, we classified 452 repetitive elements (45%) according to Wicker's classification system of TEs. These included 312 putative retrotransposons (Class I elements), 119 putative DNA transposons (Class II elements) and 21 other elements (potential host genes). Class I transposons included 110 LTR, 7 DIRS, 8 PLE, 6 LARD, 20 TRIM, 137 LINE, 11 SINE and 13 undetermined elements. Class II transposons included 87 TIR, 3 MITE, 12 Crypton, 5 Helitron, 1 Maverick and 11 undetermined elements. Potential host genes included for example a large fraction of the mitochondrial genome, various proteins, zinc finger domains, RNA binding and recognition motifs, and a herpesvirus tegument protein. Among the unclassified 553 repetitive elements, a blastx search implemented in blast2go matched 180 elements to various host gene proteins, one herpesvirus‐homolog, one transcription factor IIIA‐like element (*GTF3A*) as well as 12 retrovirus‐like polyproteins (*pol; gag‐pol*).

**Figure 2:**
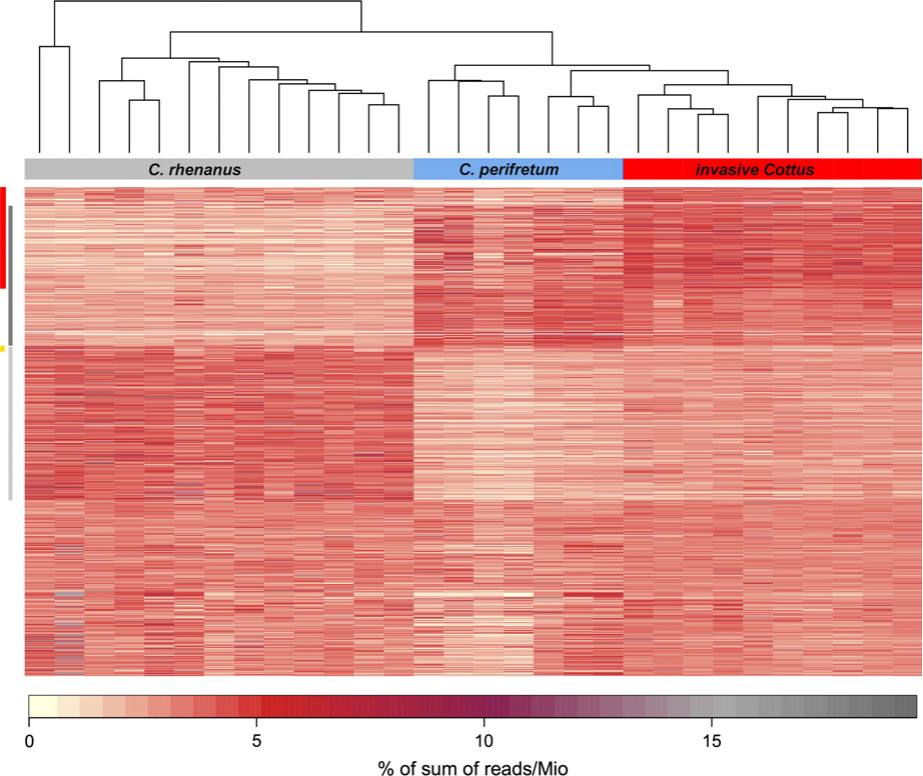
Heatmap showing mapping results of genomic reads from 30 *Cottus* genomes against 1,005 repetitive elements. Colour‐coding represents the percentage of sum of reads (number of mapped reads per Mio reads) for each repetitive element. Coloured sidebars on the left indicate 208 repetitive elements with significantly higher numbers of mapped reads in invasive *Cottus* compared to the parental species (red), 12 elements with significantly lower numbers in invasive *Cottus* than in the parental species (orange), as well as repetitive elements that are significantly higher in *C. rhenanus* (light grey), and significantly lower in *C. rhenanus* (dark grey) compared to combined invasive *Cottus* and *C. perifretum*

To assess whether invasive *Cottus* individuals (*n* = 10) showed higher copy numbers compared to both parental species (*n* = 20), we applied Wilcoxon rank‐sum tests to compare individual count data for each of 1,005 repetitive elements (one‐tailed; FDR corrected). Among all 1,005 repetitive elements, we found significant copy‐number increases in 208 (20.7%) repetitive elements with an average copy‐number increase of 30.5% compared to the parental mean (Figure 2; marked by red sidebar). In contrast, significant decreases in copy number were rare, affecting only 12 (0.01%) repetitive elements (nine unclassified elements, two putative retrotransposons, one potential host gene). Overall, increased repetitive elements showed a strong bias towards those being present in higher copy numbers in *C. perifretum* compared to *C. rhenanus* (Figure 2). This was also evident when comparing copy numbers between *C. perifretum* and *C. rhenanus* for the 208 candidate elements, revealing 112 elements (53.8%) with significantly higher copy numbers in *C. perifretum* and three elements (1.4%) with significantly higher copy numbers in *C. rhenanus* (one‐tailed Wilcoxon rank‐sum tests; FDR corrected; *p *< .05).

Out of the 208 candidate elements, 86 (41.3%) could be classified using pasteclassifier. These included 76 putative retrotransposons (Class I elements: 18 LTR, 2 DIRS, 4 PLE, 1 LARD, 7 TRIM, 38 LINE, 3 SINE), 19 putative DNA transposons (Class II elements: 12 TIR, 1 MITE, 2 Crypton elements) and 1 potential host gene (*EFHD2*). From the remaining 122 unclassified candidate elements, 27 showed blastx matches to various proteins such as *GTF3A* (induces transcription of 5S rRNA), interferon‐induced 44 protein (a putative response to hepatitis virus) and *G2E3* (a ubiquitin‐protein ligase).

Among the 86 classified candidate transposons, 15 were marked as “complete” by the pasteclassifier software, which included mostly non‐LTR retrotransposons (11 LINE, 1 SINE, 2 PLE, 1 TIR elements). No significant overrepresentation of either Class I or Class II elements could be detected among candidate transposons that were increased in invasive *Cottus* (Fisher's exact test; two‐sided; *p* = .264). When subdividing Class I elements further into LTR‐ and non‐LTR retrotransposons, elements classified to order showed a nonsignificant (Fisher's exact test; two‐sided; *p* = .053) bias towards non‐LTR retrotransposons.

Overall, the proportion of increased elements in invasive *Cottus* (20.7%) was similar between classified transposons and unclassified elements. Of 452 repetitive elements classified to transposon‐order, 86 (19%) were increased in invasive *Cottus* (retrotransposons: 24.4%; DNA transposons: 16%). Among 553 unclassified repetitive elements, 122 (22.1%) were increased in invasive *Cottus,* and out of 180 unclassified repetitive elements with some blastx match; 27 (15%) were increased in invasive *Cottus*.

### 3.4 Validation of CNV candidates and screen for de novo CNVs in F2 crosses

**Figure 3:**
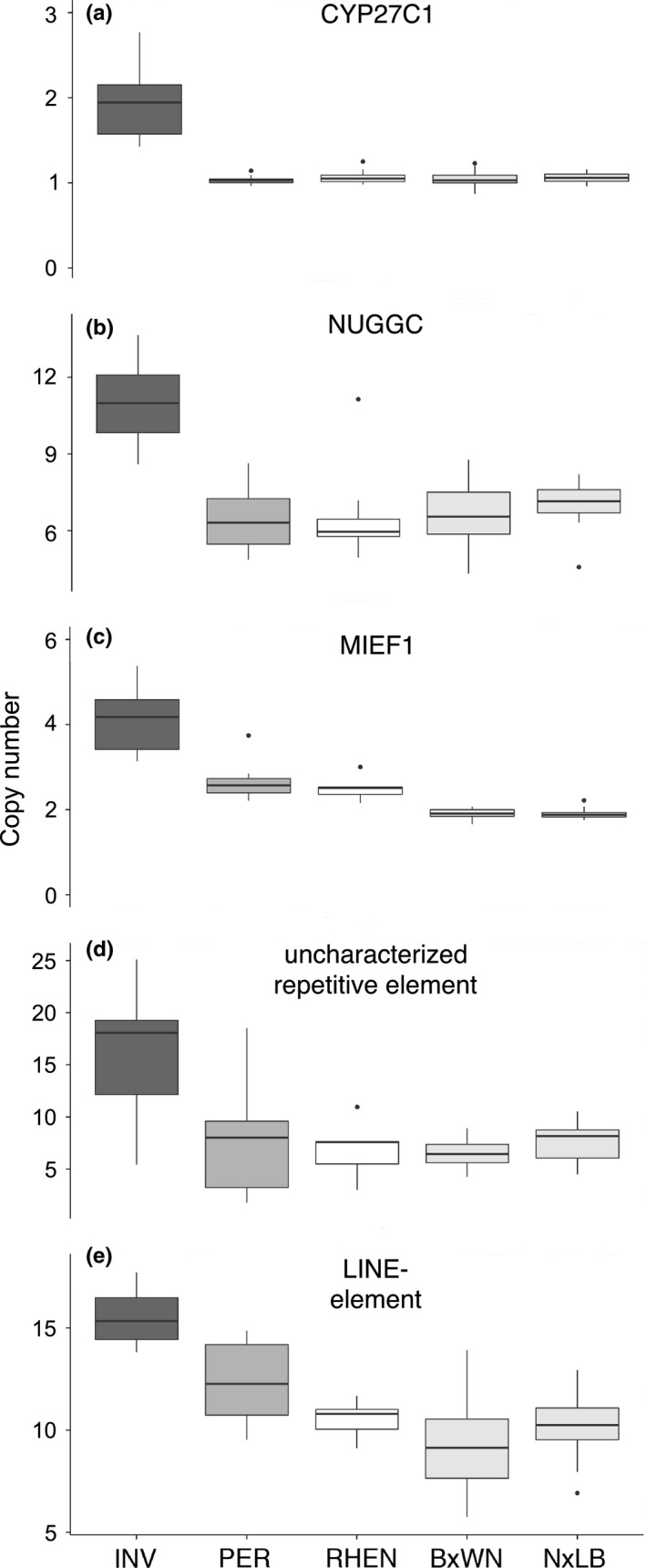
Digital droplet PCR (ddPCR) validations of copy‐number variations candidates for three genes (a–c) and two repetitive elements (d, e). Box plots show copy‐number estimates for invasive *Cottus* (INV), *C. perifretum* (PER), *C. rhenanus* (RHEN) as well as two F_2_ offspring families from laboratory crosses between *C. rhenanus* and *C. perifretum* (BxWN = Bröl × Witte Nete; NxLB = Naaf × Larse Beek)

We used ddPCR to validate increased copy numbers in invasive *Cottus* for three selected CNV candidate genes (*MIEF1, NUGGC, CYP27C1*) and three repetitive elements (one LINE element, two uncharacterized elements). These CNV candidates showed some of the strongest signals of copy‐number increases in invasive *Cottus* and were therefore chosen as a proof of principle to confirm our alternative approaches to detecting copy‐number variants. In the parental species, ddPCR revealed *CYP27C1* as a single‐copy gene, whereas *MIEF1* occurred mostly in two copies and *NUGGC* was present as a more variable multicopy gene with most individuals showing five to seven copies (Figure 3).

Because the *CYP27C1* gene expression candidate probe from Czypionka et al. (2012) was located outside exonic regions, we additionally tested a newly designed probe located within (putative) exon 8 to test for a duplication of the protein‐coding gene. Increased copy numbers could be confirmed (ANOVA; followed by Tukey post hoc tests; *p* < .05) for the three genes (including both exonic and nonexonic probes of *CYP27C1*), the LINE element and one uncharacterized repetitive element (Figure 3). One uncharacterized element (“Contig_926”) showed similar high copy numbers in invasive *Cottus* and *C. perifretum*. Among two of the increased genes (*MIEF1, NUGGC*) and the two increased repetitive elements, single individuals of either of the parental species showed similarly high copy numbers as observed in invasive *Cottus* (Figure 3).

Candidates with strong signals in our analyses were considered useful to test for de novo mutations in controlled crosses. To explore a possible role of rapidly evolving de novo structural variants, we screened 32 individuals from each of two F_2_ families (*C. rhenanus* × *C. perifretum*) for CNV of validated candidate genes (*MIEF1, NUGGC, CYP27C1*) and repetitive elements (one LINE element, one uncharacterized element) (Figure 3). None of the examined CNV candidates showed signs of de novo copy‐number increases compared to the parental species (Figure 3).

## 4 Discussion

Copy‐number variation has long been recognized to play a role in the evolution of phenotypic novelty. However, the possible contribution of structural variations to the evolution of admixed lineages remains largely unexplored in natural systems. Here, we report copy‐number increases of candidate genes (exons) as well as a proliferation of repetitive elements in a homoploid hybrid lineage of *Cottus*. The origin of invasive *Cottus* is most likely postglacial and the biogeographic settings suggest an age of <200 generations (Nolte et al., 2005). Hence, copy number increases that are specific to invasive *Cottus* could have evolved within a relatively short time frame following admixture. Herein, we found no evidence for de novo structural mutations in three genes and two repetitive elements in two families of laboratory F_2_ crosses between the parental species. However, this limited set of 64 F_2_ offspring only represents an upper bound for the frequency of such mutations in early generation hybrid crosses. It is still possible that de novo mutations occurred in natural hybrid populations at a frequency that could not be detected here. On the other hand, our ability to distinguish whether CNVs in admixed lineages are recruited from standing genetic variation or from de novo mutations is limited because of uncertainty about the variation present in natural populations. We discuss evolutionary processes that may be relevant to explain copy‐number changes in invasive *Cottus* and how they could be involved in adaptive evolution.

### 4.1 Exon copy‐number variation in admixed lineages

Although copy‐number variation between the parental species occurs frequently, we found only a very low proportion of copy number variable exons that may have increased in invasive *Cottus*. This is not surprising given the presumably recent origin of invasive *Cottus* and considering that gene duplication rates are typically slow (Katju & Bergthorsson, 2013; Lynch & Conery, 2000). It is also consistent with the general assumption that new gene duplications are quickly removed from the genome by purifying selection (Katju & Bergthorsson, 2013; Lynch & Conery, 2000; Ohno, 1970). However, the occurrence of gene duplicates that reach high frequencies in invasive *Cottus* is conspicuous. Genomic admixture in hybrids can increase structural mutation rates through retrotransposition (Michalak, 2009) or nonallelic homologous recombination events, which can be promoted by decreased genomic stability particularly in regions with a high density of tandemly arrayed repeats (Janoušek, Karn, & Laukaitis, 2013). Accordingly, copy number increases of some genes that are present in multiple copies in the parental species could be explained through nonallelic homologous recombination between tandemly arrayed gene copies. This could be true for *NUGGC*, which occurs in variable multicopy numbers in the parental *Cottus* species. A tandem organization of *NUGGC* copies appears likely because this can also be observed in the genome of the closely related threespine stickleback (UCSC Genome Browser). A larger segmental duplication could have played a role in the duplication of *MIEF1*. For this gene, conservation of synteny between *Cottus* and stickleback (Cheng, Czypionka, & Nolte, 2013) infers *si:ch211‐256m1.8* as the most likely neighbouring gene, which shows a joint increase of copy number in invasive *Cottus*. Increased copy numbers could also originate from standing genetic variation already present in populations or individuals of the parental species not sampled here. This seems plausible for genes such as *MIEF1* or *NUGGC*, for which ddPCR identified single individuals of the parental species with increased copy numbers. Hence, our study did not yield conclusive evidence that genomic admixture caused de novo copy number increases of coding genes in invasive *Cottus*.

While we cannot rule out genetic drift, the occurrence of high‐frequency gene duplicates that are close to fixation in invasive *Cottus* but not the parental species indicates a possible role of selection. A recent effort to disentangle the relative role of drift and selection suggested that about half of the high‐frequency gene duplicates in *Drosophila melanogaster* were fixed by selection and the other half by drift (Cardoso‐Moreira et al., 2016). Assuming lower effective population sizes in *Cottus*, this could mean a higher proportion of gene duplicates being fixed by drift. However, the time to fixation should be 4*Ne* under neutral expectations (e.g., Innan & Kondrashov, 2010), which in the case of 200 generations assumed for invasive *Cottus* would translate into a *Ne* of 50 that seems unrealistically low. Drift is therefore unlikely to explain all gene duplicates, and instead, some of the gene duplicates may have risen to high frequencies through selection (e.g., Feulner et al., 2013; Kondrashov, 2012). Selection could favour new alternative splice variants following duplications of exons (Abascal, Tress, & Valencia, 2015) or act on gene dosage effects that are thought to underlie most of the reported adaptive gene duplicates (Kondrashov, 2012). Increased gene dosage appears to be a possible consequence of duplications in a *GTF2IRD2*‐like gene and two members of the cytochrome P450 gene family (*CYP27C1, CYP2K1*). These genes are among the strongest candidates for increased copy numbers in our aCGH analyses and were overexpressed in juvenile stages of invasive *Cottus* in a previous gene expression study (Czypionka et al., 2012). Functions associated with cytochrome P450 genes are highly diverse (Danielson, 2002) and have been related for example to insecticide resistance (Wondji et al., 2009), adaptation to desert environments (Jirimutu et al., 2012) or immune responses to bacterial and virus infections (Iizuka et al., 2004). In teleost fishes, *CYP2K1* appears to be mostly expressed in liver and kidney and involved in detoxification of environmental pollutants (e.g., Buhler, Zhao, Yang, Miranda, & Buhler, 1997; Uno, Ishizuka, & Itakura, 2012). Enright et al. (2015) reported that *CYP27C1* may red‐shift the spectral sensitivity in zebrafish by converting vitamin A_1_ into A_2_, which translates into an enhanced behavioural response to long‐wavelength light. If *CYP27C1* has a similar function in *Cottus*, increased gene dosage could be beneficial for enhanced vision in more turbid environments (Fain, 2015) such as the larger streams inhabited by invasive *Cottus*. Finally, *GTF2IRD2* might affect the development of skeletal muscle during the growth period of juvenile stages, with increased gene dosage leading to a higher proportion of slow‐type muscle fibres (Palmer et al., 2012). Changed proportions of muscle fibre types could be involved in the acclimation to different temperature regimes by invasive *Cottus*, resembling what has been reported for other teleost fishes (e.g., Woytanowski & Coughlin, 2013). Altogether, we found some gene duplicates with potentially adaptive gene dosage effects in an evolutionary young homoploid hybrid species, but their low number and the possibility that they were recruited from standing genetic variation of the parental species does not suggest a major role of increased de novo evolution of structural variants.

### 4.2 Increases of repetitive elements

We detected a significant increase of copy numbers in 20.7% of repetitive elements including putative transposons in invasive *Cottus*. The frequency of these copy‐number increases exceeded what we observed with protein‐coding genes by far. Their proliferation could have been be induced by a “genomic shock” following exposure to environmental stress or hybridization (Belyayev, 2014; Fontdevila, 2005; McClintock, 1984; Senerchia, Felber, & Parisod, 2015). A weak bias towards non‐LTR retrotransposons among increased TEs is in line with such a burst of transposition, but this excess is not significant and other repetitive elements have increased almost equally. For example, we find increased copy numbers of putative DNA transposons that use a cut‐and‐paste mechanism and should only rarely increase in copy number. Similarly, a range of uncharacterized repetitive elements that are not likely to multiply via transposition also increased. Hence, the accumulation of repetitive elements in invasive *Cottus* cannot be fully explained through transposition. Resembling our results, transposition bursts are not evident in F_1_ hybrid sunflowers or contemporary hybrid zones between their parental species (Kawakami et al., 2011; Renaut et al., 2014). This has led to argue that a combination of hybridization and environmental stress during colonization of extreme habitats (deserts, salt marshes) could have initiated bursts of transposition in hybrid sunflower species (Kawakami et al., 2011), although subsequent experimental tests failed to induce TE proliferation under various stress conditions (Ungerer & Kawakami, 2013). The absence of transposition bursts has also been reported from retrotransposons in polyploid plant hybrids, which instead emphasized the importance of TE silencing through reorganization of methylation states and deletions of TE loci (e.g., Senerchia et al., 2015, 2016). Accordingly, an accumulation of TEs through transposition activities may be rare and is presumably limited to only a small fraction of TEs that escape silencing mechanisms without provoking strong deleterious effects (Parisod et al., 2010).

As an underexplored alternative, increased copy numbers of repetitive elements could be explained through multiple segmental duplications in centromeric or pericentromeric regions, which typically harbour large arrays of satellite repeats and TEs (Horvath et al., 2005; Ma & Jackson, 2006). Centromeres have been suggested as a primary target for genomic destabilization following interspecific hybridization in marsupial hybrids, which was shown to cause amplification of satellite and TEs (Metcalfe et al., 2007). Likewise, a massive accumulation of retro TEs has occurred in pericentromeric regions of homoploid hybrid species of sunflowers (Staton, Ungerer, & Moore, 2009). Interestingly, most of the repetitive elements increased in invasive *Cottus* have higher copy numbers in *C. perifretum* compared to *C. rhenanus* (Figure 3). Conversely, repetitive elements that occur in higher copy numbers in *C. rhenanus* compared to *C. perifretum* have rarely expanded in the invasive *Cottus* genome. This asymmetric pattern of repetitive element amplification correlates with an overrepresentation of *C. perifretum* ancestry in the genome of invasive *Cottus,* which contributes ~60% to the hybrid gene pool (Stemshorn et al., 2011). Thus, a nonrandom mechanism of repetitive element amplification with respect to ancestry appears possible. Centromere drive could quickly create such an asymmetry though a transmission advantage of longer centromeres during female meiosis, mediated by a higher number of microtubule binding sites during the segregation of chromosomes (Henikoff, Ahmad, & Malik, 2001). Such a transmission bias could produce an asymmetric pattern of repetitive element accumulation if amplified elements in invasive *Cottus* are indeed located in centromeric regions. If true, enlarged centromeres from *C. perifretum* but not *C. rhenanus* could sweep through the hybrid population, thus promoting a rapid accumulation of repetitive elements through subsequent segmental duplications or other recombination‐based mechanisms. Although speculative at present, centromere drive could commonly affect the evolution of admixed genomes, including the build‐up of reproductive isolation (e.g., Trier, Hermansen, Sætre, & Bailey, 2014) or the ancestry‐biased accumulation of repetitive elements (this study) during hybrid speciation.

## 5 Conclusions

Copy‐number variation in natural populations can contribute to the raw material for evolutionary change. The occurrence of high‐frequency gene duplicates with potentially adaptive gene dosage effects in invasive *Cottus* is in line with such a scenario but appears to be relatively rare in this study. We speculate that centromere drive followed by segmental duplications might contribute to the conspicuous accumulation of repetitive elements. While this appears to be attributable to hybridization and genomic admixture, it also suggests that it may be particularly pronounced in gene poor regions around the centromeres. On the one hand, this means that the massive copy number increase is unlikely to affect a large number of coding genes, while on the other hand, it makes it plausible that the large number of new repetitive element copies was not removed by selection. This warrants more studies that distinguish whether increased transposition rates or transposition‐independent mechanisms cause the accumulation of copy numbers in hybrid lineages and, more broadly, whether this may lead to rapid evolution of hybrid species.

## Data Accessibility

Microarray raw data, including the aCGH calibration data, were archived at the Gene Expression Omnibus database under Accession no GSE93065 (http://www.ncbi.nlm.nih.gov/geo/). Raw sequence reads of *Cottus* genomes were archived at the European Nucleotide Archive (http://www.ebi.ac.uk/ena/) under Accession nos ERR1894831 to ERR1894860 (project Accession: PRJEB20064).

## Author Contributions

S.D. and A.N.W. designed the experiments, analysed the data and wrote the manuscript. J.A. conducted the Illumina sequencing, and F.S. helped with all steps of the genome assembly and genome mapping. E.I. and X.L. contributed to the analysis of expression changes of genes and copy‐number changes of repetitive elements. All authors revised and approved the final manuscript.

## References

[mec14134-bib-0001] Abascal, F. , Tress, M. L. , & Valencia, A. (2015). The evolutionary fate of alternatively spliced homologous exons after gene duplication. Genome Biology and Evolution, 7, 1392–1403. 25931610 10.1093/gbe/evv076 PMC4494069

[mec14134-bib-0002] Abbott, R. , Albach, D. , Ansell, S. , Arntzen, J. W. , Baird, S. J. E. , Bierne, N. , … Zinner, D. (2013). Hybridization and speciation. Journal of Evolutionary Biology, 26, 229–246. 23323997 10.1111/j.1420-9101.2012.02599.x

[mec14134-bib-0003] Amos, W. (2010). Heterozygosity and mutation rate: Evidence for an interaction and its implications. BioEssays, 32, 82–90. 19967709 10.1002/bies.200900108

[mec14134-bib-0004] Bateson, W. (1909). Heredity and variation in modern lights In Seward A. C. (Ed.), Darwin and modern science (pp. 85–101). Cambridge: CUP.

[mec14134-bib-0005] Belyayev, A. (2014). Bursts of transposable elements as an evolutionary driving force. Journal of Evolutionary Biology, 27, 2573–2584. 25290698 10.1111/jeb.12513

[mec14134-bib-0006] Benjamini, Y. , & Hochberg, Y. (1995). Controlling the false discovery rate: A practical and powerful approach to multiple testing. Journal of the Royal Statistical Society. Series B (Methodological), 57, 289–300.

[mec14134-bib-0007] Bivand, R. , & Gebhardt, A. (2000). Implementing functions for spatial statistical analysis using the R language. Journal of Geographical Systems, 2, 307–317.

[mec14134-bib-0008] Bonchev, G. , & Parisod, C. (2013). Transposable elements and microevolutionary changes in natural populations. Molecular Ecology Resources, 13, 765–775. 23795753 10.1111/1755-0998.12133

[mec14134-bib-0009] Buhler, J. L. W. , Zhao, X. , Yang, Y. H. , Miranda, C. L. , & Buhler, D. R. (1997). Expression of a constitutive cytochrome P450 (CYP2K1) in livers of rainbow trout (*Oncorhynchus mykiss*) embryo and sac‐fry. Aquatic Toxicology, 37, 237–251.

[mec14134-bib-0010] Cardoso‐Moreira, M. , Arguello, J. R. , Gottipati, S. , Harshman, L. G. , Grenier, J. K. , & Clark, A. G. (2016). Evidence for the fixation of gene duplications by positive selection in *Drosophila* . Genome Research, 26, 787–798. 27197209 10.1101/gr.199323.115 PMC4889967

[mec14134-bib-0011] Casacuberta, E. , & González, J. (2013). The impact of transposable elements in environmental adaptation. Molecular Ecology, 22, 1503–1517. 23293987 10.1111/mec.12170

[mec14134-bib-0012] Casals, F. , Cáceres, M. , & Ruiz, A. (2003). The Foldback‐like transposon Galileo is involved in the generation of two different natural chromosomal inversions of *Drosophila buzzatii* . Molecular Biology and Evolution, 20, 674–685. 12679549 10.1093/molbev/msg070

[mec14134-bib-0013] Castel, S. E. , & Martienssen, R. A. (2013). RNA interference in the nucleus: Roles for small RNAs in transcription, epigenetics and beyond. Nature Reviews Genetics, 14, 100–112. 10.1038/nrg3355 PMC4205957 23329111

[mec14134-bib-0014] Cheng, J. , Czypionka, T. , & Nolte, A. W. (2013). The genomics of incompatibility factors and sex determination in hybridizing species of *Cottus* . Heredity, 111, 520–529. 23981957 10.1038/hdy.2013.76 PMC3833689

[mec14134-bib-0015] Chuong, E. B. , Elde, N. C. , & Feschotte, C. (2016). Regulatory evolution of innate immunity through co‐option of endogenous retroviruses. Science, 351, 1083–1087. 26941318 10.1126/science.aad5497 PMC4887275

[mec14134-bib-0016] Conesa, A. , Götz, S. , García‐Gómez, J. M. , Terol, J. , Talón, M. , & Robles, M. (2005). Blast2GO: A universal tool for annotation, visualization and analysis in functional genomics research. Bioinformatics, 21, 3674–3676. 16081474 10.1093/bioinformatics/bti610

[mec14134-bib-0017] Czypionka, T. , Cheng, J. , Pozhitkov, A. , & Nolte, A. W. (2012). Transcriptome changes after genome‐wide admixture in invasive sculpins (*Cottus*). Molecular Ecology, 21, 4797–4810. 22650446 10.1111/j.1365-294X.2012.05645.x

[mec14134-bib-0018] Danielson, P. B. (2002). The cytochrome P450 superfamily: Biochemistry, evolution and drug metabolism in humans. Current Drug Metabolism, 3, 561–597. 12369887 10.2174/1389200023337054

[mec14134-bib-0019] Dhami, P. , Coffey, A. J. , Abbs, S. , Vermeesch, J. R. , Dumanski, J. P. , Woodward, K. J. , … Vetrie, D. (2005). Exon array CGH: Detection of copy‐number changes at the resolution of individual exons in the human genome. American Journal of Human Genetics, 76, 750–762. 15756638 10.1086/429588 PMC1199365

[mec14134-bib-0020] Dobzhansky, T. (1937). Genetics and the origin of species. New York: Columbia University.

[mec14134-bib-0021] Enright, J. M. , Toomey, M. B. , Sato, S. Y. , Temple, S. E. , Allen, J. R. , Fujiwara, R. , … Corbo, J. C. (2015). Cyp27c1 red‐shifts the spectral sensitivity of photoreceptors by converting vitamin A_1_ into A_2_ . Current Biology, 25, 3048–3057. 26549260 10.1016/j.cub.2015.10.018 PMC4910640

[mec14134-bib-0022] Fain, G. L. (2015). Phototransduction: Making the chromophore to see through the murk. Current Biology, 25, R1126–R1142. 26654369 10.1016/j.cub.2015.10.004

[mec14134-bib-0023] Feulner, P. G. D. , Chain, F. J. J. , Panchal, M. , Eizaguirre, C. , Kalbe, M. , Lenz, T. , … Bornberg‐Bauer, E. (2013). Genome‐wide patterns of standing genetic variation in a marine population of three‐spined sticklebacks. Molecular Ecology, 22, 635–649. 22747593 10.1111/j.1365-294X.2012.05680.x

[mec14134-bib-0024] Finn, R. D. , Coggill, P. , Eberhardt, R. Y. , Eddy, S. R. , Mistry, J. , Mitchell, A. L. , … Bateman, A. (2016). The Pfam protein families database: Towards a more sustainable future. Nucleic Acids Research, 44, 279–285. 10.1093/nar/gkv1344 PMC4702930 26673716

[mec14134-bib-0025] Fontdevila, A. (2005). Hybrid genome evolution by transposition. Cytogenetic and Genome Research, 110, 49–55. 16093657 10.1159/000084937

[mec14134-bib-0026] García Guerreiro, M. P. (2014). Interspecific hybridization as a genomic stressor inducing mobilization of transposable elements in *Drosophila* . Mobile Genetic Elements, 4, e34394. 25136509 10.4161/mge.34394 PMC4132227

[mec14134-bib-0027] García Guerreiro, M. P. (2015). Changes of Osvaldo expression patterns in germline of male hybrids between the species *Drosophila buzzatii* and *Drosophila koepferae* . Molecular Genetics and Genomics, 290, 1471–1483. 25711309 10.1007/s00438-015-1012-z

[mec14134-bib-0028] Grabherr, M. G. , Haas, B. J. , Yassour, M. , Levin, J. Z. , Thompson, D. A. , Amit, I. , … Regev, A. (2011). Full‐length transcriptome assembly from RNA‐seq data without a reference genome. Nature Biotechnology, 15, 644–652. 10.1038/nbt.1883 PMC3571712 21572440

[mec14134-bib-0029] Henikoff, S. , Ahmad, K. , & Malik, H. S. (2001). The centromere paradox: Stable inheritance with rapidly evolving DNA. Science, 293, 1098–1102. 11498581 10.1126/science.1062939

[mec14134-bib-0030] Hibbeler, S. , Scharsack, J. P. , & Becker, S. (2008). Housekeeping genes for quantitative expression studies in the three‐spined stickleback *Gasterosteus aculeatus* . BMC Molecular Biology, 9, 18. 18230138 10.1186/1471-2199-9-18 PMC2254436

[mec14134-bib-0031] Hoede, C. , Arnoux, S. , Moisset, M. , Chaumier, T. , Inizan, O. , Jamilloux, V. , & Quesneville, H. (2014). PASTEC: An automatic transposable element classification tool. PLoS ONE, 9, e91929. 24786468 10.1371/journal.pone.0091929 PMC4008368

[mec14134-bib-0032] Horvath, J. E. , Gulden, C. L. , Vallente, R. U. , Eichler, M. Y. , Ventura, M. , McPherson, J. D. , … Eichler, E. E. (2005). Punctuated duplication seeding events during the evolution of human chromosome 2p11. Genome Research, 15, 914–927. 15965031 10.1101/gr.3916405 PMC1172035

[mec14134-bib-0033] Iizuka, N. , Oka, M. , Hamamoto, Y. , Mori, N. , Tamesa, T. , Tangoku, A. , … Yamada‐Okabe, H. (2004). Altered levels of cytochrome P450 genes in hepatitis B or C virus‐infected liver identified by oligonucleotide microarray. Cancer Genomics & Proteomics, 1, 53–58. 31394618

[mec14134-bib-0034] Innan, H. , & Kondrashov, F. (2010). The evolution of gene duplications: Classifying and distinguishing between models. Nature Reviews Genetics, 11, 97–108. 10.1038/nrg2689 20051986

[mec14134-bib-0035] Janoušek, V. , Karn, R. C. , & Laukaitis, C. M. (2013). The role of retrotransposons in gene family expansions: Insights from the mouse *Abp* gene family. BMC Evolutionary Biology, 13, 107. 23718880 10.1186/1471-2148-13-107 PMC3669608

[mec14134-bib-0036] Jirimutu, W. Z. , Ding, G. , Chen, G. , Sun, Y. , Zhang, H. , Wang, L. , … Meng, H. (2012). Genome sequences of wild and domestic bactrian camels. Nature Communications, 3, 1202. 10.1038/ncomms2192 PMC3514880 23149746

[mec14134-bib-0037] Jurka, J. , Kapitonov, V. V. , Pavlicek, A. , Klonowski, P. , Kohany, O. , & Walichiewicz, J. (2005). Repbase Update, a database of eukaryotic repetitive elements. Cytogenetic and Genome Research, 110, 462–467. 16093699 10.1159/000084979

[mec14134-bib-0038] Katju, V. , & Bergthorsson, V. (2013). Copy‐number changes in evolution: Rates, fitness effects and adaptive significance. Frontiers in Genetics, 4, 273. 24368910 10.3389/fgene.2013.00273 PMC3857721

[mec14134-bib-0039] Kawakami, T. , Dhakal, P. , Katterhenry, A. N. , Heatherington, C. A. , & Ungerer, M. C. (2011). Transposable element proliferation and genome expansion are rare in contemporary sunflower hybrid populations despite widespread transcriptional activity of LTR retrotransposons. Genome Biology and Evolution, 3, 156–167. 21282712 10.1093/gbe/evr005 PMC3048363

[mec14134-bib-0040] Kent, W. J. (2002). BLAT – The BLAST‐like alignment tool. Genome Research, 12, 656–664. 11932250 10.1101/gr.229202 PMC187518

[mec14134-bib-0041] Kofler, R. , Orozco‐terWengel, P. , De Maio, N. , Pandey, R. V. , Nolte, V. , Futschik, A. , … Schlötterer, C. (2011). Popoolation: A toolbox for population genetic analysis of next generation sequencing data from pooled individuals. PLoS ONE, 6, e15925. 21253599 10.1371/journal.pone.0015925 PMC3017084

[mec14134-bib-0042] Kondrashov, F. A. (2012). Gene duplication as a mechanism of genomic adaptation to a changing environment. Proceedings of the Royal Society of London B: Biological Sciences, 279, 5048–5057. 10.1098/rspb.2012.1108 PMC3497230 22977152

[mec14134-bib-0043] Labrador, M. , & Fontdevila, A. (1994). High transposition rates of Osvaldo a new *Drosophila buzzatii* retrotransposon. Molecular and General Genetics MGG, 245, 661–674. 7545973 10.1007/BF00297273

[mec14134-bib-0044] Landry, C. R. , Hartl, D. L. , & Ranz, J. M. (2007). Genome clashes in hybrids: Insights from gene expression. Heredity, 99, 483–493. 17687247 10.1038/sj.hdy.6801045

[mec14134-bib-0045] Lexer, C. , Lai, Z. , & Rieseberg, L. H. (2003). Candidate gene polymorphisms associated with salt tolerance in wild sunflower hybrids: Implications for the origin of *Helianthus paradoxus*, a diploid hybrid species. New Phytologist, 161, 225–233. 10.1046/j.1469-8137.2003.00925.x PMC2601661 19079642

[mec14134-bib-0046] Lynch, M. , & Conery, J. S. (2000). The evolutionary fate and consequences of duplicate genes. Science, 290, 1151–1155. 11073452 10.1126/science.290.5494.1151

[mec14134-bib-0047] Ma, J. , & Jackson, S. A. (2006). Retrotransposon accumulation and satellite amplification mediated by segmental duplication facilitate centromere expansion in rice. Genome Research, 16, 251–259. 16354755 10.1101/gr.4583106 PMC1361721

[mec14134-bib-0048] Machado, H. E. , Jui, G. , Joyce, D. A. , Reilly, C. R. L. III , Lunt, D. H. , & Renn, S. C. P. (2014). Gene duplication in an African cichlid adaptive radiation. BMC Genomics, 15, 161. 24571567 10.1186/1471-2164-15-161 PMC3944005

[mec14134-bib-0049] Maheshwari, S. , & Barbash, D. A. (2011). The genetics of hybrid incompatibilities. Annual Review of Genetics, 45, 331–355. 10.1146/annurev-genet-110410-132514 21910629

[mec14134-bib-0050] Mallet, J. (2007). Hybrid speciation. Nature, 446, 279–283. 17361174 10.1038/nature05706

[mec14134-bib-0051] McClintock, B. (1984). The significance of responses of the genome to challenge. Science, 226, 792–801. 15739260 10.1126/science.15739260

[mec14134-bib-0052] McDermott, G. P. , Do, D. , Litterst, C. M. , Maar, D. , Hindson, C. M. , Steenblock, E. R. , … Lowe, A. J. (2013). Multiplexed target detection using DNA‐binding dye chemistry in droplet digital PCR. Analytical Chemistry, 85, 11619–11627. 24180464 10.1021/ac403061n

[mec14134-bib-0053] Metcalfe, C. J. , Bulazel, K. V. , Ferreri, G. C. , Schroeder‐Reiter, E. , Wanner, G. , Rens, W. , … O'Neill, R. J. O. (2007). Genomic instability within centromeres of interspecific marsupial hybrids. Genetics, 177, 2507–2517. 18073443 10.1534/genetics.107.082313 PMC2219476

[mec14134-bib-0054] Michalak, P. (2009). Epigenetic, transposon and small RNA determinants of hybrid dysfunctions. Heredity, 102, 45–60. 18545265 10.1038/hdy.2008.48

[mec14134-bib-0055] Miotke, L. , Lau, B. T. , Rumma, R. T. , & Ji, H. P. (2014). High sensitivity detection and quantitation of DNA copy number and single nucleotide variants with single color droplet digital PCR. Analytical Chemistry, 86, 2618–2624. 24483992 10.1021/ac403843j PMC3982983

[mec14134-bib-0056] Müller, H. J. (1942). Isolating mechanisms, speciation, and temperature. Biological Symposia, 6, 71–125.

[mec14134-bib-0057] Nolte, A. W. , Freyhof, J. , Stemshorn, K. C. , & Tautz, D. (2005). An invasive lineage of sculpins, *Cottus sp*. (Pisces, Teleostei) in the Rhine with new habitat adaptations has originated from hybridization between old phylogeographic groups. Proceedings of the Royal Society of London B: Biological Sciences, 272, 2379–2387. 10.1038/rspb.2005.3231 PMC1559961 16243698

[mec14134-bib-0058] Nolte, A. W. , Freyhof, J. , & Tautz, D. (2006). When invaders meet locally adapted types: Rapid moulding of hybrid zones between sculpins (*Cottus*, Pisces) in the Rhine system. Molecular Ecology, 15, 1983–1993. 16689913 10.1111/j.1365-294X.2006.02906.x

[mec14134-bib-0059] Nolte, A. W. , Gompert, Z. , & Buerkle, C. A. (2009). Variable patterns of introgression in two sculpin hybrid zones suggest that genomic isolation differs among populations. Molecular Ecology, 18, 2615–2627. 19457191 10.1111/j.1365-294X.2009.04208.x

[mec14134-bib-0060] Nolte, A. W. , & Tautz, D. (2010). Understanding the onset of hybrid speciation. Trends in Genetics, 26, 54–58. 20044166 10.1016/j.tig.2009.12.001

[mec14134-bib-0061] Ohno, S. (1970). Evolution by gene duplication. New York, NY: Springer.

[mec14134-bib-0062] O'Neill, R. J. W. , O'Neill, M. J. , & Graves, J. A. M. (1998). Undermethylation associated with retroelement activation and chromosome remodelling in an interspecific mammalian hybrid. Nature, 393, 68–72. 9590690 10.1038/29985

[mec14134-bib-0063] Orozco, L. D. , Cokus, S. J. , Ghazalpour, A. , Ingram‐Drake, L. , Wang, S. , van Nas, A. , … Lusis, A. J. (2009). Copy number variation influences gene expression and metabolic traits in mice. Human Molecular Genetics, 18, 4118–4129. 19648292 10.1093/hmg/ddp360 PMC2758141

[mec14134-bib-0064] Palmer, S. J. , Taylor, K. M. , Santucci, N. , Widagdo, J. , Chan, Y. K. A. , Yeo, J. L. , … Hardeman, E. C. (2012). GTF2IRD2 from the Williams‐Beuren critical region encodes a mobile‐element‐derived fusion protein that antagonizes the action of its related family members. Journal of Cell Science, 125, 5040–5050. 22899722 10.1242/jcs.102798 PMC3533390

[mec14134-bib-0065] Papp, B. , Pal, C. , & Hurst, L. D. (2003). Dosage sensitivity and the evolution of gene families in yeast. Nature, 424, 194–197. 12853957 10.1038/nature01771

[mec14134-bib-0066] Parisod, C. , Alix, K. , Just, J. , Petit, M. , Sarilar, V. , Mhiri, C. , … Grandbastien, M. A. (2010). Impact of transposable elements on the organization and function of allopolyploid genomes. New Phytologist, 186, 37–45. 20002321 10.1111/j.1469-8137.2009.03096.x

[mec14134-bib-0067] Pozhitkov, A. E. , Noble, P. A. , Bryk, J. , & Tautz, D. (2014). A revised design for microarray experiments to account for experimental noise and uncertainty of probe response. PLoS ONE, 9, e91295. 24618910 10.1371/journal.pone.0091295 PMC3949741

[mec14134-bib-0068] Renaut, S. , Nolte, A. , & Bernatchez, L. (2010). Mining transcriptome sequences towards identifying adaptive single nucleotide polymorphisms in lake whitefish species pairs (*Coregonus spp*. Salmonidae). Molecular Ecology, 19, 115–131. 20331775 10.1111/j.1365-294X.2009.04477.x

[mec14134-bib-0069] Renaut, S. , Rowe, H. C. , Ungerer, M. C. , & Rieseberg, L. H. (2014). Genomics of homoploid hybrid speciation: Diversity and transcriptional activity of long terminal repeat retrotransposons in hybrid sunflowers. Philosophical Transactions of the Royal Society B, 369, 20130345. 10.1098/rstb.2013.0345 PMC4071519 24958919

[mec14134-bib-0070] Rieseberg, L. H. (1997). Hybrid origins of plant species. Annual Review of Ecology, Evolution, and Systematics, 28, 359–389.

[mec14134-bib-0071] Rieseberg, L. H. , Archer, M. A. , & Wayne, R. K. (1999). Transgressive segregation, adaptation and speciation. Heredity, 83, 363–372. 10583537 10.1038/sj.hdy.6886170

[mec14134-bib-0072] Ritchie, M. E. , Phipson, B. , Wu, D. , Hu, Y. , Law, C. W. , Shi, W. , & Smyth, G. K. (2015). limma powers differential expression analyses for RNA‐sequencing and microarray studies. Nucleic Acids Research, 43, e47 https://doi.org/10.1093/nar/gkv007 25605792 10.1093/nar/gkv007 PMC4402510

[mec14134-bib-0073] Scavetta, R. J. , & Tautz, D. (2010). Copy number changes of CNV regions in intersubspecific crosses of the house mouse. Molecular Biology and Evolution, 27, 1845–1856. 20200126 10.1093/molbev/msq064

[mec14134-bib-0074] Schumer, M. , Rosenthal, G. G. , & Andolfatto, P. (2014). How common is homoploid hybrid speciation? Evolution, 68, 1553–1560. 24620775 10.1111/evo.12399

[mec14134-bib-0075] Sedlazeck, F. J. , Rescheneder, P. , & von Haeseler, A. (2013). NextGenMap: Fast and accurate read mapping in highly polymorphic genomes. Bioinformatics, 29, 2790–2791. 23975764 10.1093/bioinformatics/btt468

[mec14134-bib-0076] Senerchia, N. , Felber, F. , North, B. , Sarr, A. , Guadagnuolo, R. , & Parisod, C. (2016). Differential introgression and reorganization of retrotransposons in hybrid zones between wild wheats. Molecular Ecology, 25, 2518–2528. 26678573 10.1111/mec.13515

[mec14134-bib-0077] Senerchia, N. , Felber, F. , & Parisod, C. (2015). Genome reorganization in F_1_ hybrids uncovers the role of retrotransposons in reproductive isolation. Proceedings of the Royal Society of London B: Biological Sciences, 282, 20142827. 10.1098/rspb.2014.2874 PMC4375867 25716787

[mec14134-bib-0078] Song, L. , Florea, L. , & Langmead, B. (2014). Lighter: Fast and memory‐efficient sequencing error correction without counting. Genome Biology, 15, 509. 25398208 10.1186/s13059-014-0509-9 PMC4248469

[mec14134-bib-0079] Staton, S. E. , Ungerer, M. C. , & Moore, R. C. (2009). The genomic organization of Ty3/Gypsy‐like retrotransposons in *Helianthus* (Asteraceae) homoploid hybrid species. American Journal of Botany, 96, 1646–1655. 21622351 10.3732/ajb.0800337

[mec14134-bib-0080] Stemshorn, K. C. , Reed, F. A. , Nolte, A. W. , & Tautz, D. (2011). Rapid formation of distinct hybrid lineages after secondary contact of two fish species (*Cottus* sp.). Molecular Ecology, 20, 1475–1491. 21251111 10.1111/j.1365-294X.2010.04997.x

[mec14134-bib-0081] Symer, D. E. , Connelly, C. , Szak, S. T. , Caputo, E. M. , Cost, G. J. , Parmigiani, G. , & Boeke, J. D. (2002). Human L1 retrotransposition is associated with genetic instability in vivo. Cell, 110, 327–338. 12176320 10.1016/s0092-8674(02)00839-5

[mec14134-bib-0082] Thorvaldsdóttir, H. , Robinson, J. T. , & Mesirov, J. P. (2013). Integrative Genomics Viewer (IGV): High‐performance genomics data visualization and exploration. Briefings in Bioinformatics, 14, 178–192. 22517427 10.1093/bib/bbs017 PMC3603213

[mec14134-bib-0083] Trier, C. N. , Hermansen, J. S. , Sætre, G. P. , & Bailey, R. I. (2014). Evidence for mito‐nuclear and sex‐linked reproductive barriers between the hybrid Italian Sparrow and its parent species. PloS Genetics, 10, e1004075. 24415954 10.1371/journal.pgen.1004075 PMC3886922

[mec14134-bib-0084] Ungerer, M. C. , & Kawakami, T. (2013). Transcriptional dynamics of LTR retrotransposons in early generation and ancient sunflower hybrids. Genome Biology and Evolution, 5, 329–337. 23335122 10.1093/gbe/evt006 PMC3590766

[mec14134-bib-0085] Ungerer, M. C. , Strakosh, S. C. , & Stimpson, K. M. (2009). Proliferation of Ty3/gypsy‐like retrotransposons in hybrid sunflower taxa inferred from phylogenetic data. BMC Biology, 7, 40. 19594956 10.1186/1741-7007-7-40 PMC2715380

[mec14134-bib-0086] Uno, T. , Ishizuka, M. , & Itakura, T. (2012). Cytochrome P450 (CYP) in fish. Environmental Toxicology and Pharmacology, 34, 1–13. 22418068 10.1016/j.etap.2012.02.004

[mec14134-bib-0087] Vogt, J. , Bengesser, K. , Claes, K. B. M. , Wimmer, K. , Mautner, V. F. , van Minkelen, R. , … Kehrer‐Sawatzki, H. (2014). SVA retrotransposon insertion‐associated deletion represents a novel mutational mechanism underlying large genomic copy number changes with non‐recurrent breakpoints. Genome Biology, 15, R80. 24958239 10.1186/gb-2014-15-6-r80 PMC4229983

[mec14134-bib-0088] Wicker, T. , Sabot, F. , Hua‐Van, A. , Bennetzen, J. L. , Capy, P. , Chalhoub, B. , … Schulman, A. H. (2007). A unified classification system for eukaryotic transposable elements. Nature Reviews Genetics, 8, 973–982. 10.1038/nrg2165 17984973

[mec14134-bib-0089] Wondji, C. S. , Irving, H. , Morgan, J. , Lobo, N. F. , Collins, F. H. , Hunt, R. H. , … Ranson, H. (2009). Two duplicated P450 genes are associated with pyrethroid resistance in *Anopheles funestus*, a major malaria vector. Genome Research, 19, 452–459. 19196725 10.1101/gr.087916.108 PMC2661802

[mec14134-bib-0090] Woytanowski, J. R. , & Coughlin, D. J. (2013). Thermal acclimation in rainbow smelt, *Osmerus mordax*, leads to faster myotomal muscle contractile properties and improved swimming performance. Biology Open, 2, 343–350. 23519555 10.1242/bio.20133509 PMC3603416

[mec14134-bib-0091] Xie, Z. , Wang, L. , Wang, L. , Wang, Z. , Lu, Z. , Tian, D. , … Hurst, L. D. (2016). Mutation rate analysis via parent‐progeny sequencing of the perennial peach. I. A low rate in woody perennials and a higher mutagenicity in hybrids. Proceedings of the Royal Society of London B: Biological Sciences, 283, 20161016. 10.1098/rspb.2016.1016 PMC5095371 27798292

[mec14134-bib-0092] Xing, J. , Wang, H. , Belancio, V. P. , Cordaux, R. , Deininger, P. L. , & Batzer, M. A. (2006). Emergence of primate genes by retrotransposon‐mediated sequence transduction. Proceedings of the National Academy of Sciences, 103, 17608–17613. 10.1073/pnas.0603224103 PMC1693794 17101974

[mec14134-bib-0093] Zahurak, M. , Parmigiani, G. , Yu, W. , Scharpf, R. B. , Berman, D. , Schaeffer, E. , … Cope, L. (2007). Pre‐processing Agilent microarray data. BMC Bioinformatics, 8, 142. 17472750 10.1186/1471-2105-8-142 PMC1876252

[mec14134-bib-0094] Zytnicki, M. , Akhunov, E. , & Quesneville, H. (2014). Tedna: A transposable element de novo assembler. Bioinformatics, 30, 2656–2658. 24894500 10.1093/bioinformatics/btu365

