# Supplementary material for: Copy number increases of transposable elements and protein‐coding genes in an invasive fish of hybrid origin
Source: Mol Ecol. 2017 Apr 28;26(18):4712–24. doi: 10.1111/mec.14134 (PMC5638112; doi:10.1111/mec.14134)
Supplement: Supplementary file 1 [file MEC-26-4712-s001.docx]

SUPPORTING INFORMATION

**Copy number increases of transposable elements and protein coding genes in an invasive fish of hybrid origin**

Stefan Dennenmoser^1,5^, Fritz J. Sedlazeck^2^, Elzbieta Iwaszkiewicz^1^, Xiang-Yi Li^3^, Janine Altmüller^4^, Arne W. Nolte^1,5^

^1^Department for Evolutionary Genetics, Max-Planck Institute for Evolutionary Biology, August Thienemann Strasse 2, 24306 Plön, Germany

^2^Johns Hopkins University, Department of Computer Science, Baltimore, 21218 MD, USA

^3^University of Zurich, Department of Evolutionary Biology and Environmental Studies, Winterthurerstrasse 190, CH-8057 Zurich

^4^Cologne Center for Genomics, and Institute of Human Genetics, University of Cologne, 50931 Cologne, Germany

^5^Carl von Ossietzky University Oldenburg, Institute for Biology, Carl von Ossietzky Str. 9-11, 26129 Oldenburg, Germany

Table of Contents:

**Table S1** Primer sequences and digital droplet PCR (ddPCR) protocols used for validation of copy number candidates.

**Table S2** Functional descriptions of aCGH copy number candidate genes that were found to be increased or decreased in invasive *Cottus* compared to the parental species *C. rhenanus* and *C. perifretum*.

**Supplemental Table S1**: Primer sequences and digital droplet PCR (ddPCR) protocols used for validation of copy number candidates.

| Gene | Primer (5’ – 3’) | Tm |
| --- | --- | --- |
| *RPL13A*  (141 bp) | Left: AGGAAGGAGAAGGCTAAGCT  Right: ACCAGCTCAAACAAGGACTC | 52-58°C |
| *MIEF1*  (72 bp) | Left: cgcaaagggaagaaagatgac  Right: cacaagcttggcattggaaa | 58°C |
| *NUGGC*  (56 bp) | Left: atcttcctggaaatggggac  Right: taccgttttccacatcttgt | 54°C |
| *CYP27C1*_exon  (64 bp) | Left: ggccctttgtcactattcca  Right: tcgaaagtctgaagcatcgg | 54°C |
| *CYP27C1*  (57 bp) | Left: ttgcaggaaagtttgatggc  Right: tcaatatgtagggctggattca | 54°C |
| Contig_127  (65 bp) | Left: TTTGGGAGCAAGAGTGTCTG  Right: AGCAGAACTTAAGAGAGCGC | 52°C |
| Contig_926  (64 bp) | Left: TGGCAATCAACTTTTCCCCA  Right: ACTGTTTGGTGAAAAGAAAGGC | 54°C |
| Contig_296  LINE-element  (74 bp) | Left: CAACAGAGGCAGCCAAACTA  Right: GGACCAGCTTGTGAAACAGT | 54°C |
|  |  |  |

The 24 µl mastermix contained 12 µl 2x Qx200 EvaGreen ddPCR supermix, 5-10 µl of the diluted DNA sample (~1-5 ng DNA), 50 nM of the target primers, 200 nM of the reference (*rpl13a*) primers, and water. Two cutting enzymes (Csp6I and FspBI, using 5 U of enzyme per 1 µg of DNA) were added to the ddPCR mastermix for a digestion time of 10 minutes. For all PCR reactions, 20 µl of the mastermix was mixed with 70 µl Droplet Generation Oil (Bio-Rad Laboratories, CA, USA), and partitioned into droplets using a QX100 Droplet Generator (Bio-Rad). Cycling conditions for ddPCR amplification followed a 2-step cycling protocol with a ramp speed of 2.5°C/sec.: 95°C for 5 min, 40 cycles of 95°C for 30s, 52°-58°C for 60s, followed by 4°C for 5 min, and 90°C for 5 min. For all PCR products, fluorescence signals of individual droplets were measured using an upgraded QX100 Droplet Digital reader (Bio-Rad), and analyzed with the QuantaSoft droplet reader software (Bio-Rad).

**Supplemental Table S2:** Functional descriptions of aCGH copy number candidate genes that were found to be increased or decreased in invasive *Cottus* compared to the parental species *C. rhenanus* and *C. perifretum*.

| **Gene** | **Function** (UniProtKB / Swiss-Prot / Entrez Gene) |
| --- | --- |
| ***Increased in invasive Cottus*** | |
| *MIEF1* | Mitochondrial Elongation Factor1. Mitochondrial outer membrane protein which regulates mitochondrial fission. Promotes the recruitment and association of the fission mediator dynamin-related protein 1 (DNM1L) to the mitochondrial surface independently of the mitochondrial fission FIS1 and MFF proteins. Regulates DNM1L GTPase activity and DNM1L oligomerization. Binds ADP and can also bind GDP, although with lower affinity. Does not bind CDP, UDP, ATP, AMP or GTP. Inhibits DNM1L GTPase activity in the absence of bound ADP. Requires ADP to stimulate DNM1L GTPase activity and the assembly of DNM1L into long, oligomeric tubules with a spiral pattern, as opposed to the ring-like DNM1L oligomers observed in the absence of bound ADP. Does not require ADP for its function in recruiting DNM1L. |
| *SI:CH211-256M1.8* | Uncharacterized protein. |
| *NUGGC* | Nuclear GTPase, germinal center associated. Plays a role as replication-related GTPase protein in germinal center B-cell. |
| *TENC1B (TNS2)* | Tensin like C1 domain containing phosphatase b. Regulates cell motility and proliferation. May have phosphatase activity. Reduces AKT1 phosphorylation. Lowers AKT1 kinase activity and interferes with AKT1 signaling. |
| *RC3H2* | Ring finger and CCCH-type domains 2. Post-transcriptional repressor of mRNAs containing a conserved stem loop motif, called constitutive decay element (CDE), which is often located in the 3-UTR, as in HMGXB3, ICOS, IER3, NFKBID, NFKBIZ, PPP1R10, TNF and in many more mRNAs (By similarity). Binds to CDE and promotes mRNA deadenylation and degradation. This process does not involve miRNAs (By similarity). In follicular helper T (Tfh) cells, represses of ICOS and TNFRSF4 expression, thus preventing spontaneous Tfh cell differentiation, germinal center B-cell differentiation in the absence of immunization and autoimmunity (By similarity). In resting or LPS-stimulated macrophages, controls inflammation by suppressing TNF expression (By similarity). Also recognizes CDE in its own mRNA and in that of paralogous RC3H2, possibly leading to feedback loop regulation (By similarity). |
| *GTF2IRD2* | General Transcription Factor II-I Repeat Domain-Containing Protein 2A. This gene is one of several closely related genes on chromosome 7 encoding proteins containing helix-loop-helix motifs. These proteins may function as regulators of transcription. The encoded protein is unique in that its C-terminus is derived from CHARLIE8 transposable element sequence. This gene is located in a region of chromosome 7 that is deleted in Williams-Beuren syndrome, and loss of this locus may contribute to the cognitive phenotypes observed in this disease. |
| *MPP5A* | Membrane protein, palmitoylated 5a. This gene encodes a member of the p55-like subfamily of the membrane-associated guanylate kinase (MAGUK) gene superfamily. The encoded protein participates in the polarization of differentiating cells, has been shown to regulate myelinating Schwann cells (PMID: 20237282), and is one of the components of the Crumbs complex in the retina. Mice which express lower levels of the orthologous protein have retinal degeneration and impaired vision (PMID: 22114289). May play a role in tight junctions biogenesis and in the establishment of cell polarity in epithelial cells. May modulate SC6A1/GAT1-mediated GABA uptake by stabilizing the transporter. Required for localization of EZR to the apical membrane of parietal cells and may play a role in the dynamic remodeling of the apical cytoskeleton (By similarity). |
| *RALGAPA1* | Ral GTPase Activating Protein, Alpha Subunit 1. Catalytic subunit of the heterodimeric RalGAP1 complex which acts as a GTPase activator for the Ras-like small GTPases RALA and RALB. |
| *GALNT7* | GalNAc transferase 7. This gene encodes GalNAc transferase 7, a member of the GalNAc-transferase family. The enzyme encoded by this gene controls the initiation step of mucin-type O-linked protein glycosylation and transfer of N-acetylgalactosamine to serine and threonine amino acid residues. This enzyme is a type II transmembrane protein and shares common sequence motifs with other family members. Unlike other family members, this enzyme shows exclusive specificity for partially GalNAc-glycosylated acceptor substrates and shows no activity with non-glycosylated peptides. This protein may function as a follow-up enzyme in the initiation step of O-glycosylation. |
| *PCGF1* | Polycomb group ring finger 1. PCGF1 is a mammalian homolog of the *Drosophila* polycomb group genes, which act as transcriptional repressors to regulate anterior-posterior patterning in early embryonic development. |
| *CYP2K1* | Cytochrome P450, member 2K1. Belongs to the cytochrome P450 family. |
| *CYP27C1* | Cytochrome P450, member 27C1. This gene encodes a member of the cytochrome P450 superfamily of enzymes. The cytochrome P450 proteins are monooxygenases, which catalyze many reactions involved in drug metabolism and synthesis of cholesterol, steroids and other lipids. |
| ***Decreased in Invasive Cottus*** | |
| *ZGC:73345* | Reactive oxygen species modulator 1. Has antibacterial activity against a variety of bacteria including *S.aureus, P.aeruginosa* and *M.tuberculosis*. Acts by inducing bacterial membrane breakage (By similarity). Induces production of reactive oxygen species (ROS), which are necessary for cell proliferation. May play a role in inducing oxidative DNA damage and replicative senescence. May play a role in the coordination of mitochondrial morphology and cell proliferation (By similarity). |
| *SCL39A3* | Solute carrier family 39. Acts as a zinc-influx transporter. |
| *DMC1* | DNA Meiotic Recombinase 1. This gene encodes a member of the superfamily of recombinases (also called DNA strand-exchange proteins). Recombinases are important for repairing double-strand DNA breaks during mitosis and meiosis. This protein, which is evolutionarily conserved, is reported to be essential for meiotic homologous recombination and may thus play an important role in generating diversity of genetic information. |
| *FKBP2* | FK506 Binding Protein 2, 13kDa . The protein encoded by this gene is a member of the immunophilin protein family, which play a role in immunoregulation and basic cellular processes involving protein folding and trafficking. This encoded protein is a cis-trans prolyl isomerase that binds the immunosuppressants FK506 and rapamycin. It is thought to function as an ER chaperone and may also act as a component of membrane cytoskeletal scaffolds. Multiple alternatively spliced variants, encoding the same protein, have been identified. |
| *MYT1L* | Myelin Transcription Factor 1-Like. May function as a panneural transcription factor associated with neuronal differentiation. May play a role in the development of neurons and oligodendrogalia in the CNS (By similarity). |
| *DR1* | Down-Regulator Of Transcription 1. The association of the DR1/DRAP1 heterodimer with TBP results in a functional repression of both activated and basal transcription of class II genes. This interaction precludes the formation of a transcription-competent complex by inhibiting the association of TFIIA and/or TFIIB with TBP. Can bind to DNA on its own. Component of the ATAC complex, a complex with histone acetyltransferase activity on histones H3 and H4. |
|  | |
| *TLX1* | T-Cell Leukemia Homeobox 1. This gene encodes a nuclear transcription factor that belongs to the NK-linked or NK-like (NKL) subfamily of homeobox genes. The encoded protein is required for normal development of the spleen during embryogenesis. This protein is also involved in specification of neuronal cell fates. Ectopic expression of this gene due to chromosomal translocations is associated with certain T-cell acute lymphoblastic leukemias. |
| *RNF2* | Ring Finger Protein 2. E3 ubiquitin-protein ligase that mediates monoubiquitination of Lys-119 of histone H2A (H2AK119Ub), thereby playing a central role in histone code and gene regulation. H2AK119Ub gives a specific tag for epigenetic transcriptional repression and participates in X chromosome inactivation of female mammals. May be involved in the initiation of both imprinted and random X inactivation. Essential component of a Polycomb group (PcG) multiprotein PRC1-like complex, a complex class required to maintain the transcriptionally repressive state of many genes, including Hox genes, throughout development. PcG PRC1 complex acts via chromatin remodeling and modification of histones, rendering chromatin heritably changed in its expressibility. E3 ubiquitin-protein ligase activity is enhanced by BMI1/PCGF4. Acts as the main E3 ubiquitin ligase on histone H2A of the PRC1 complex, while RING1 may rather act as a modulator of RNF2/RING2 activity. In resting B- and T-lymphocytes, interaction with AURKB leads to block its activity, thereby maintaining transcription in resting lymphocytes. |
| *ZNF574* | Zinc Finger Protein 574. May be involved in transcriptional regulation. |
| *SLC17A8* | Solute Carrier Family 17, Member 8. This gene encodes a vesicular glutamate transporter. The encoded protein transports the neurotransmitter glutamate into synaptic vesicles before it is released into the synaptic cleft. |
| *SETD5* | SET Domain Containing 5. This function of this gene has yet to be determined but mutations in this gene have been associated with autosomal dominant mental retardation-23. |
| *VDAC1* | Outer Mitochondrial Membrane Protein Porin 1. This gene encodes a voltage-dependent anion channel protein that is a major component of the outer mitochondrial membrane. The encoded protein facilitates the exchange of metabolites and ions across the outer mitochondrial membrane and may regulate mitochondrial functions. This protein also forms channels in the plasma membrane and may be involved in transmembrane electron transport. |
| *NUDT4A* | Nudix (Nucleoside Diphosphate Linked Moiety X)-Type Motif 4a. The protein encoded by this gene regulates the turnover of diphosphoinositol polyphosphates. The turnover of these high-energy diphosphoinositol polyphosphates represents a molecular switching activity with important regulatory consequences. Molecular switching by diphosphoinositol polyphosphates may contribute to regulating intracellular trafficking. |
